# Rapid Discrimination for Traditional Complex Herbal Medicines from Different Parts, Collection Time, and Origins Using High-Performance Liquid Chromatography and Near-Infrared Spectral Fingerprints with Aid of Pattern Recognition Methods

**DOI:** 10.1155/2015/727589

**Published:** 2015-08-09

**Authors:** Haiyan Fu, Yao Fan, Xu Zhang, Hanyue Lan, Tianming Yang, Mei Shao, Sihan Li

**Affiliations:** The Modernization Engineering Technology Research Center of Ethnic Minority Medicine of Hubei Province, College of Pharmacy, South-Central University for Nationalities, Wuhan 430074, China

## Abstract

As an effective method, the fingerprint technique, which emphasized the whole compositions of samples, has already been used in various fields, especially in identifying and assessing the quality of herbal medicines. High-performance liquid chromatography (HPLC) and near-infrared (NIR), with their unique characteristics of reliability, versatility, precision, and simple measurement, played an important role among all the fingerprint techniques. In this paper, a supervised pattern recognition method based on PLSDA algorithm by HPLC and NIR has been established to identify the information of *Hibiscus mutabilis* L. and *Berberidis radix*, two common kinds of herbal medicines. By comparing component analysis (PCA), linear discriminant analysis (LDA), and particularly partial least squares discriminant analysis (PLSDA) with different fingerprint preprocessing of NIR spectra variables, PLSDA model showed perfect functions on the analysis of samples as well as chromatograms. Most important, this pattern recognition method by HPLC and NIR can be used to identify different collection parts, collection time, and different origins or various species belonging to the same genera of herbal medicines which proved to be a promising approach for the identification of complex information of herbal medicines.

## 1. Introduction

Herbal medicines, with their effective pharmacological functions, low toxicity, and less side effects, have been widely used for thousands of years all over the world, which are becoming more and more popular and promising type of medicines [1–5]. However, the same species of herbal medicines with different growing conditions can have some different chemical constituents and pharmacological activities, and what is more, even different parts of the same herbal may considerably differ in types and quantities of chemical components [6–8]. Thus, the quality assessment and control of herbal medicines are an important concern for both the health authorities and the public [9–11]. The traditional methods such as visible inspection and thin-layer chromatography either are subjective in the aspect of nature or require experienced users, resulting in scarcely meeting the criteria needed to support their use widely. Therefore, a faster, more accurate, and sensitive identification method is required to determine the herbal medicines.

The large majority of investigations only focused on several pharmacologically active constituents to assess the quality and potency of the herbal medicine, which barely represent the whole quality of herbal medicines [12, 13]. The fingerprint technique, which emphasized the whole compositions of samples and focuses on identifying and assessing the quality of the samples, was admitted by WHO, State Food and Drug Administration (SFDA) of China (2000), and other authorities as a strategy for quality assessment of herbal medicines [14]. Due to its reliability, versatility, and precision, high-performance liquid chromatography (HPLC) played an important role among all the fingerprint techniques [15–17]. In recent years, HPLC method with various detectors has been developed for qualitative and quantitative analysis of various herbal medicines, such as* Angelica dahurica* [18],* Panax ginseng* [19], and* Berberidis radix* [20]. If the peaks are well separated, a herbal sample can be authenticated by chemical fingerprinting based on the presence or absence of characteristic peaks. However, when coming to identifying a large number of similar samples, numerous variables (peaks) may not be efficiently discriminated. In addition, the traditional HPLC fingerprints cannot meet the requirements of high throughput analysis due to the low column efficiency and long analysis time with generally more than 1 hr. In recent years, near-infrared (NIR) as having characteristics of high efficiency, low cost, simple measurement, little sample preparation, quick data analysis, and nondestructive analytical technique has been widely used in several scientific fields, especially in the identification of herbal medicines [21–24]. Nonetheless, just as HPLC, the fingerprint information provided by NIR spectra may be difficult to interpret. So establishing effective and robust chemometrics methods has been extensively concerned with. For example, Woo's team used Mahalanobis distance and discriminant PLS2 combined with NIR spectroscopy to discriminate herbal medicines according to geographical origin [25], Frizon used the principal component analysis (PCA) and the partial least squares (PLS) regression to determine the total phenolic compounds in yerba mate by NIR [26], and Segtnan's research studied the structure of water using two-dimensional near-infrared correlation spectroscopy [27]. Unfortunately, these researches all failed to reach the completely correct recognition.

Considering that wavelengths of near-infrared reflectance spectra set as the explanatory variable can be hundreds or even thousands, which often had further quantity than the number of samples, multiple correlation was caused by this; ordinary pattern recognition methods like PCA or LDA are difficult to use to create an effective mathematical model. For another, by using PLSDA, the spectral data can be compressed to a lower-dimensional spatial data [28]. Through reasonable selection of the main components and removing interfering components and main ingredients of interference factors, only useful principal components can be involved in linear regression, which can perfectly solve the problem of prediction results being confused. Thus, PLSDA model can be much better to relate the dummy code for the full NIR original and preprocessing spectral variables of herbal medicines [29, 30].

In this study, traditional fingerprint of chromatograms combined with PLSDA and three classical chemometrics methods, as PCA, LDA, and PLSDA model with different fingerprint preprocessing of NIR spectra variables, are adopted to extract and discriminate otherness of different collection parts, collection time, and different origins of* Hibiscus mutabilis* L. and different species of* Berberidis radix*. Other than PCA and LDA that can merely have good learning performance and do well in the training sets, PLSDA model shows good performance in the area of identification of herbal medicines and can be perfectly employed in the analysis of the influence of different collection parts, collection time, and different origins or various species belonging to the same genera of herbal medicines by both chromatograms and NIR spectra. What is more, this is the first time for a supervised pattern recognition method based on PLSDA algorithm by HPLC or NIR to identify the sheer amount of information of herbal medicines at one time.

## 2. Material and Methods

### 2.1. Collection and Identification of Raw Materials

*Hibiscus mutabilis* L. leaves and stems samples were purchased in Anhui, collected in Nanning, Guangxi, or South-Central University For Nationalities while* Berberis soulieana* Schneid (BSS),* Berberis henryana* Schneid (BHS),* Berberis gagnepainii* Schneid (BGS), and* Berberis triacanthophora* Fedde (BTF) samples were collected from Jianshi, Wuhan, between May and June in 2012. All the samples were identified by Professor Dingrong Wan.

### 2.2. Apparatus

Antaris II FT-NIR spectrometer, Result 3.0 spectral collecting software (Thermo Electron Co., USA), UltiMate 3000 analytical HPLC (Dionex Co., USA), DZF-6021 vacuum oven (Shanghai Yiheng Technical Co., Ltd.), and FW135 herbal grinder (Tianjin Taisite Instrument Co., Ltd.) were used.

### 2.3. Sample Preparation

*Hibiscus mutabilis* L. samples used in HPLC were crushed with herbal medicine grinder, sifted by 80 mesh sieve, and set aside while* Berberidis radix* samples used in HPLC were dried in vacuum at 60°C for 24 hours, crushed with the grinder, and sifted by 40 mesh sieve for further use. All the samples used in NIR were crushed with the grinder and sifted into fine powders by 200 mesh sieve, then vacuum-dried at 60°C for 24 hours, and stored in a dryer spare.

### 2.4. HPLC Conditions

An ECOSIL 120-5-C18 SH (250 mm  ×Φ 4.6 mm, 5 *μ*m) column was used in the fingerprint analysis of* Hibiscus mutabilis* L. while a hyperchrome-HPLC-C18 (250 mm  ×Φ 4.6 mm, 5 *μ*m) column was used in the analysis of* Berberidis radix*. All analyses of* Hibiscus mutabilis* L. were performed at a column temperature of 30°C, with a mobile phase of methanol (A)-0.1% acetate aqueous solution (B) (see Table S1 of the Supplementary Material available online at http://dx.doi.org/10.1155/2015/727589), an injection volume of 10 *μ*L, and a flow rate of 1.0 mL/min. To gain the most suitable measurement wavelength, the test solution will be full-wavelength- (200–400 nm) scanned by UV. Taking into account the end absorbance of the mobile phase of methanol and maximum absorption wave of* Hibiscus mutabilis* L. test solution at 269 nm, several wavelengths with large absorbance were added to select one with more peaks and better separation. Ultimately, the UV absorbance of the eluent was set at 269 nm. On the other hand, analyses of* Berberidis radix* were performed in the same situation except with a mobile phase of methanol (A)-0.25% phosphoric acid (B) (Table S2) and the UV absorbance of the eluent was measured at 230 nm.

### 2.5. Preparation of Test Solution

In order to make it possible for fingerprint peaks to show what ingredients* Hibiscus mutabilis* L. really contains and avoid byproducts (such as hydrolysis products) and nonmedicinal ingredients interferences, petroleum ether was used to remove chlorophyll or other fat-soluble ingredients and then dried in a rotary vacuum evaporator and volume was set with methanol. By comparing the transfer rate of products using ultrasound methanol, ethanol, ultrasound, methanol water mixture reflux, ethanol water mixture reflux, and so forth, extraction methods, ethanol water mixture reflux 2 h (ethanol concentration of 60%) was taken for the highest transfer rate. Accurately weighed 2 g* Hibiscus mutabilis* L. leaves or stems sample was dissolved in 30 mL of 60% ethanol, refluxed for 2 h, and then filtered after it cooled down. The filtrate was extracted twice with 15 mL of petroleum ether. The petroleum ether layer was discarded while the aqueous alcohol layer was evaporated on a rotary instrument rotation evaporated and the residue was dissolved in methanol. After filtering, the sample was set to a volume of 25 mL in a volumetric flask and filtered by 0.22 *μ*m microporous membrane and the resulting solution was used as the test solution 1.

Compared with methanol, ethanol, and reflux in water bath and ultrasonic extraction method, hydrochloric acid-methanol (volume ratio = 1 : 50) with ultrasonic extraction was found to be the optimal choice. Accurately weighed 0.50 g* Berberidis radix* sample was dissolved in 50 mL of methanol and 1 mL of hydrochloric acid. Then the sample was weighed, ultrasonic extracted for 2 h, and cooled to room temperature. After being weighed again, methanol was added to complement the weight loss and then filtered. The filtrate was set to a volume of 100 mL by methanol in a volumetric flask. At last, 2 mL of the sample was taken and filtered by 0.22 *μ*m microporous membrane; the resulting solution was used as the test solution 2.

### 2.6. Fingerprints Preparation by HPLC

Chromatographic conditions were used as above; accurately drawn 10 *μ*L of each test solution was injected into the HPLC and recorded 72 min chromatogram.

### 2.7. Method Validation

Precision of the sample application and measurement of peak area were carried out using 5 replicates of the same sample and were expressed in terms of percent relative standard deviation (%RSD).

Stability studies: the measurement of peak area were carried out at 0, 4, 8, 16, and 24 hours, respectively, using 5 replicates of the same sample and were expressed in terms of percent relative standard deviation (%RSD).

Repeatability studies: repeatability of the sample application and measurement of peak area were carried out using 5 different samples which come from the same batch of herbs and were expressed in terms of percent relative standard deviation (%RSD).

### 2.8. Methods of Sample Measurement and Data Preprocessing by NIR

Sample powders were directly put into the quartz sample cup smoothly, and then air was used as the reference and the background was deducted. Spectra were collected by using integrating sphere diffuse reflectance with the collecting region at 10000–4000 cm^−1^ and a resolution of 8 cm^−1^. Each sample was randomly divided into four parts and scanned 32 times and 60 spectra were collected.

### 2.9. Method of Chemometrics

All original chromatograms and spectra were preprocessed by second-order derivative (2nd derivative) and multiple scatter correction (MSC). Preprocessed methods, PCA, LDA, and PLSDA programs, were written and performed using a MATLAB 2010a (Math Works, Natick, MA, USA). For PLSDA model, vector *fj* was used to encode category of the *j*th samples group, in which the element of the *j*th position was 1 and the other elements were 0. The types of unknown samples can be identified according to the dummy codes of the vectors in the classification matrix. If the maximal element of the *i*th sample appeared at the *j*th position of the classification vector, the sample will be identified as the *j*th group.

## 3. Results and Discussion

### 3.1. Fingerprints of* Hibiscus mutabilis* L. and* Berberidis radix* by HPLC

#### 3.1.1. Visual Analysis of Fingerprints

Firstly, methodological study was conducted. The %RSD of precision, stability, and repeatability study for the samples of* Hibiscus mutabilis* L. and* Berberidis radix* and measurement of peak areas was found to be less than 5%, while measurement of relative retention time was less than 3%. The %RSD indicates that the method has an acceptable level of precision.

**Figure 1:**
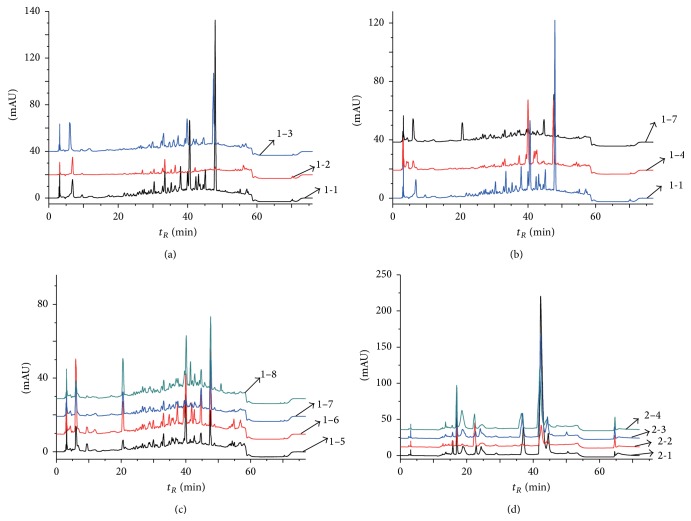
Comparison of fingerprints of different collection parts (a), collection time (b), and different origins (c) of* Hibiscus mutabilis* L. and different species of* Berberidis radix* (d) by HPLC.

In order to develop and validate an efficient method for the analysis of the influence of different collection parts, collection time, and different origins of* Hibiscus mutabilis* L., fingerprints of samples by HPLC were established. The samples correspond to the numbers in Table 1. Samples with different collection parts numbered 1-1, 1-2, and 1-3 as shown in Table 1 were made into test solutions and analyzed by the used above methods. These three fingerprints were superimposed in Figure 1(a). As were shown in the spectra, compared to the spectrum of sample 1-1, the peaks in fingerprint of 1-2 were much fewer and had a greater difference in height and peak areas in parts of some common peaks which had a lower peak height and less peak area than sample 1-1. On the other hand, compared to sample 1-1, both the peak position and peak appearance time of 1-3 were roughly the same and the only differences were the peak heights and peak areas. It reveals that big differences exist in chemical compositions and their corresponding contents of different collection parts of* Hibiscus mutabilis*, which means that confused collection parts of herbals will generate a lot of problems, such as the impact of the quality of herbals and even medication effects, which may cause major medical incidents.

When coming to the comparison of fingerprints of different origins of* Hibiscus mutabilis* L., samples 1-1, 1-4, and 1-7 with different origins as listed in Table 1 were made into test solutions and analyzed by the used above methods. These three fingerprints were superimposed in Figure 1(b). Compared to the spectrum of sample 1-1, the situations of peak appearance are quite different and worse in fingerprints of 1-4, 1-7 which had less peak shapes and different peak heights and peak areas at the same peak appearance time. This may be caused by the differences in chemical compositions and their corresponding contents of* Hibiscus mutabilis* L. leaves from different origins. As is known to all, some herbs collected in certain areas contain higher content of active ingredients which has a better quality and a more obvious treatment effect, but, instead, some herbs in this area are of poor quality. This method, however, provides an important technical basis for the identification of authentic ingredients.

Samples 1-5, 1-6, 1-7, and 1-8 with different collection time as shown in Table 1 were made into test solutions and analyzed by the used above methods to recognize different collection time of* Hibiscus mutabilis* L. According to the four fingerprints superimposed in Figure 1(c), under the same retention time, part of common peak areas and peak heights also had small differences, which proved that different collection time of* Hibiscus mutabilis* L. can lead to the variations in chemical compositions and their contents. Because the formation of chemical compositions in the plant takes place in a particular period, chemical compositions and their corresponding contents in different acquisition time also have slight differences. Either the acquisition time is earlier or later than the correct time, these active ingredients may be degraded or even transformed into other harmful substances, and the quality and safety of medicines cannot be guaranteed. Thus, this method provides a reliable way to identify collection time of herbal medicines and guarantee the better safety for patients.

In the recognition of* Berberidis radix*, to study an efficient method for the identification of various species belonging to the same genera, fingerprints of four different species of* Berberidis radix* named* Berberis soulieana* Schneid (BSS),* Berberis henryana* Schneid (BHS),* Berberis gagnepainii* Schneid (BGS), and* Berberis triacanthophora* Fedde (BTF) by HPLC were established. The samples correspond to the numbers in Table 1 and were made into test solutions and then analyzed by above methods. Fingerprints of the four samples numbered 2-1, 2-2, 2-3, and 2-4 were superimposed in Figure 1(d).

According to these 4 fingerprints, the peak heights and the peak areas were different at 8 common peaks marked in the chromatograms, which means that small chemical composition differences but big content differences exist in these four different species of* Berberidis radix*. Compared with the other three fingerprints, some peak areas and peak heights were significantly smaller in sample 2-2 at the same retention time. What is more, a common peak in the retention time between 30 min and 40 min lacked in 2-2, which certified that less types of the chemical composition and lesser content of common chemical composition were in BHS. In addition, spectrograms of 2-1 and 2-4 had similar positions of peaks appearing, peak areas, and peak heights at the same retention time, showing that chemical compositions were substantially the same in BSS and BTF but with a few differences in the content. This showed that, even if belonging to the same genera, different species of herbal medicines had their own character which were different from each other. Thus, fingerprints by HPLC for the analysis of species difference provided an efficient and reliable method to identify herbal medicines and find similar ones to replace some herbs which were unavailable.

#### 3.1.2. Fingerprints by HPLC Combined with Partial Least Squares Discriminant Analysis

To obtain a more rapid and effective identification results by HPLC, chemometric methods were required to extract useful information for the recognition of different types of* Hibiscus mutabilis* L. and* Berberidis radix*. 48 HPLC chromatograms of the test solution data for 8 groups of* Hibiscus mutabilis* L. and 24 HPLC chromatogram for 4 groups of* Berberidis radix* were randomly divided into the training set and prediction set and the optimal proportions of the training set in all the samples were selected. Detailed information about the samples from 8 groups of* Hibiscus mutabilis* L. and 4 groups of* Berberidis radix* was listed in Table 2. PLSDA model was used to relate the dummy code for the full HPLC original chromatogram variables for different sample groups. The optimal number of latent variables was, respectively, determined as 6 to obtain the least error numbers of training and prediction sets.

**Figure 2:**
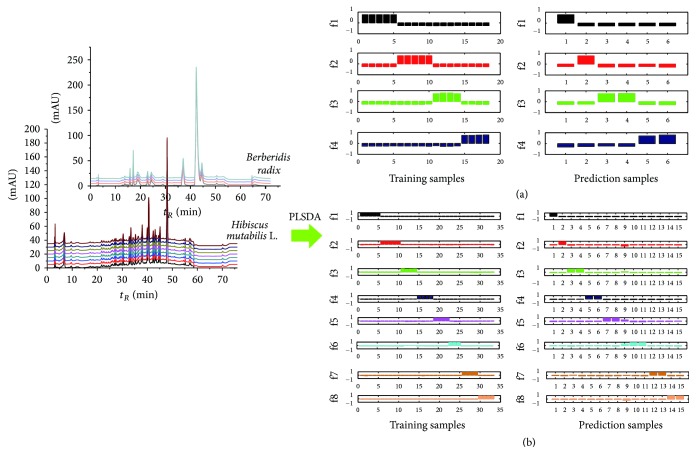
Assigned plots of dummy codes of the training set and prediction set for raw HPLC chromatogram of* Hibiscus mutabilis* L. (a) and* Berberidis radix* (b) in PLSDA model.

Figure 2 shows the assigned plots of dummy codes of the training set and prediction set for raw HPLC chromatogram in PLSDA model. Herein, the dummy codes for the 8 groups of* Hibiscus mutabilis* L. can be coded as f1 (1,0, 0,0, 0,0, 0,0), f2 (0,1, 0,0, 0,0, 0,0), f3 (0,0, 1,0, 0,0, 0,0), f4 (0,0, 0,1, 0,0, 0,0), f5 (0,0, 0,0, 1,0, 0,0), f6 (0,0, 0,0, 0,1, 0,0), f7 (0,0, 0,0, 0,0, 1,0), and f8 (0,0, 0,0, 0,0, 0,1) while four groups of* Berberidis radix* can be coded as f1 (1,0, 0,0), f2 (0,1, 0,0), f3 (0,0, 1,0), and f4 (0,0, 0,1). Samples from all groups were classified by the position of the maximal dummy codes of samples. It can be clearly seen that, no matter for* Hibiscus mutabilis* L. or* Berberidis radix*, all training samples and prediction samples belonging to all groups were identified accurately with a perfect recognition rate of 100% in both training set and prediction set. This result proved that the HPLC chromatograms combined with partial least squares discriminant analysis can be used as a new method for a fast and accurate discrimination of herbal medicines of different collection parts, collection time, and different origins of* Hibiscus mutabilis* L. or various species of* Berberidis radix*.

### 3.2. Rapid Recognition of* Hibiscus mutabilis* L. and* Berberidis radix* Using Near-Infrared Spectroscopy Combined with Chemometric Methods

**Figure 3:**
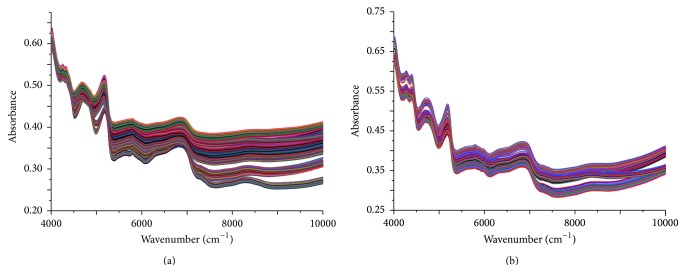
The raw NIR spectra of* Hibiscus mutabilis* L. (a) and* Berberidis radix* (b).

Although fingerprints by HPLC provided an efficient and accurate method for the identification of herbal medicines, it was too cumbersome and time-consuming. What is more, it needed professionals to operate. Thus, near-infrared spectroscopy combined with chemometric methods was used to identify herbal medicines which were different in collection parts, collection time, origins, and species. Chemical components in both* Hibiscus mutabilis* L. and* Berberidis radix* are complex that contain many types of bioactive constituents. The average NIR spectra of each group are displayed to reflect the overlay fingerprint information in Figures 3(a) and 3(b). As is shown, the raw NIR spectra were seriously overlapped, which made the accurate assignment of characteristic peaks quite difficult. This can be attributed to the contributions of multicomponents in the samples and the shifts resulted from their interactions. Therefore, chemometric methods were required to extract useful information for the recognition of* Hibiscus mutabilis* L. and* Berberidis radix* of samples.

In order to obtain better recognition results, classical chemical pattern recognition methods of principal component analysis (PCA), linear discriminant analysis (LDA), and partial least squares discriminant analysis (PLSDA) model were used to relate the dummy code for the full NIR original and preprocessing spectral variables of different sample groups.

640-sample spectra of 8 different kinds of* Hibiscus mutabilis* L. and 240-sample spectra of 4 different species of* Berberidis radix* were randomly divided into the training set and the prediction set (Table 2). The training set was used to build the model while the prediction set was used to validate it, and the discrimination results were analyzed for comparison.

**Figure 4:**
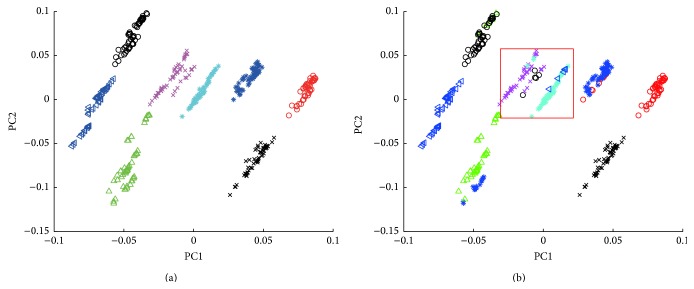
Score plots of the raw spectra by PCA of 8 different kinds of* Hibiscus mutabilis* L. samples in training sets and in prediction sets.

Firstly, PCA, known as a common method in the chemical pattern recognition which is mainly used for classification and clustering in the analytic processes of herbal identification, was used to do the principal component decomposition of spectral matrix of the samples in both training sets and prediction sets to make the new variables data structure characterize features of the original ones as much as possible. The two corresponding largest eigenvalues, that is, the score vectors of the first and the second principal components, were obtained and set as the projection-axis-to-plot to describe the spatial distribution characteristics of the samples. As is shown in Figure 4, score plots of the first and the second principal components for raw NIR spectra of 8 different kinds of* Hibiscus mutabilis* L. samples in training sets (a) can be completely distinguished and so do MSC and 2nd derivative spectra (which is not shown here), which shows that PCA training model does have relatively good learning performance. But unfortunately, those in prediction sets were confused and failed to show the correct results. On the other hand, the results of* Berberidis radix* were quite good (Figure S1). So this method cannot be used extensively to distinguish herbal medicines with a variety of mixed information.

**Figure 5:**
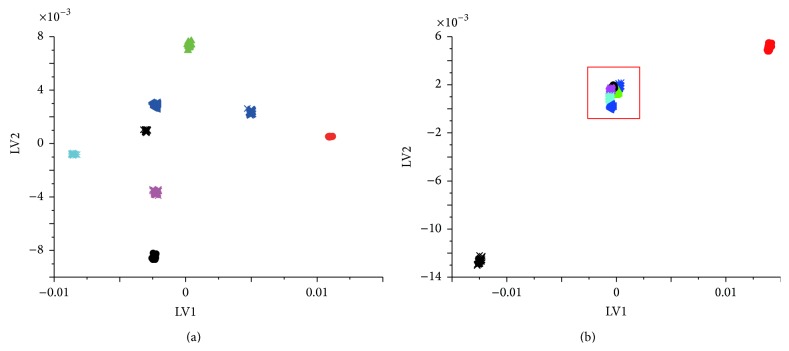
Score plots of the raw NIR spectra by LDA of 8 different kinds of* Hibiscus mutabilis* L. samples in training sets and in prediction sets.

Other than looking for the vector space that can best describe the original data like PCA, LDA was used to find the vector space that can distinguish between various types of data completely. This method can be used to extract the low-dimensional features with the most discriminating ability from the high-dimensional feature space which can gather all the samples in the same type and separate different ones. Thus, spectral matrix by NIR of the samples in both training sets and prediction sets was decomposed with LDA and the two largest eigenvalues, that is, the first and the second hidden variables, were obtained and set as the projection-axis-to-plot to describe the spatial distribution characteristics of the samples.  Figure 5 shows scores of the first and the second hidden variables for raw NIR spectra, MSC, and 2nd derivative spectra of 8 different kinds of* Hibiscus mutabilis* L. samples, respectively. Just as the results by PCA, samples in training sets can be completely distinguished (the results of MSC and 2nd derivative spectra were not listed here), while those in prediction sets (b) were confused and failed to show the correct results and so did samples of* Berberidis radix* (Figure S2). Thus, this method was also abandoned.

**Figure 6:**
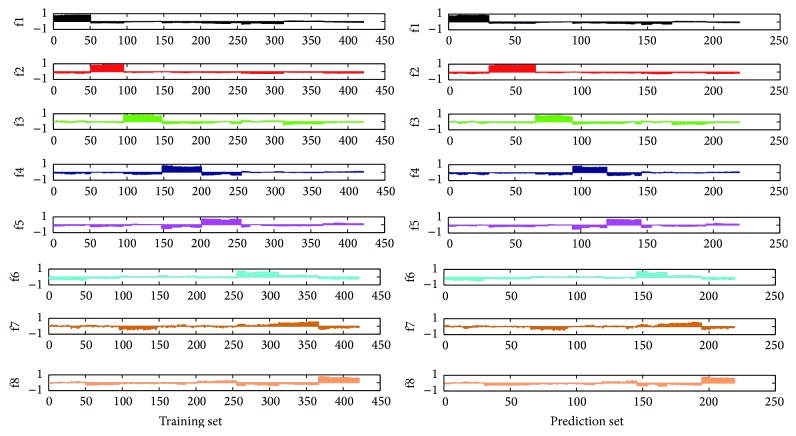
Assigned plots of dummy codes of the training set and prediction set for the raw NIR spectra in PLSDA model.

Due to the fact that the wavelengths of near-infrared spectra set as the explanatory variable had further quantity than the number of samples, ordinary pattern recognition methods were far from creating a stable, high precision mathematical model. Thus, PLSDA model was used to solve the problem of prediction results confusion and relate the dummy code for the full NIR original and preprocessing spectral variables of the 8 different* Hibiscus mutabilis* L. groups or the 4 different* Berberidis radix* groups. Table 2 showed that assigned plots of dummy codes of the training set and prediction set for raw NIR spectra in PLSDA model. Similar figures were not shown on assigned plots of dummy codes for MSC and 2nd derivative spectra in PLSDA model. Herein, the dummy codes for the 8 groups were coded as f1 (1,0, 0,0, 0,0, 0,0), f2 (0,1, 0,0, 0,0, 0,0), f3 (0,0, 1,0, 0,0, 0,0), f4 (0,0, 0,1, 0,0, 0,0), f5 (0,0, 0,0, 1,0, 0,0), f6 (0,0, 0,0, 0,1, 0,0), f7 (0,0, 0,0, 0,0, 1,0), f8 (0,0, 0,0, 0,0, 0,1) while for the 4 groups were coded as f1 (1,0, 0,0, 0,0), f2 (0,1, 0,0, 0,0), f3 (0,0, 1,0, 0,0), f4 (0,0, 0,1, 0,0), f5 (0,0, 0,0, 1,0), and f6 (0,0, 0,0, 0,1). Samples from all groups were classified by the position of the maximal dummy codes of samples. It can be clearly seen that, no matter for raw NIR spectra (Figure 6), MSC or 2nd derivative spectra (not shown here), all training samples and prediction samples belonging to all groups of* Hibiscus mutabilis* L. were identified accurately and so did* Berberidis radix* (Figure S3). Excellent forecasted results of NIR by PLSDA were obtained with all the recognition rate of 100%. A perfect recognition rate for original fingerprint, MSC fingerprint, and 2nd derivative fingerprint of NIR spectra was achieved by the PLSDA models. All the recognition rates of 100% for above three fingerprints of NIR spectra were obtained by the PLSDA models. This revealed that near-infrared spectroscopy combined with PLSDA method can be used to identify* Hibiscus mutabilis* L. and* Berberidis radix*, which were different in collection parts, collection time, origins, and species. What is more, it provided a more rapid, efficient, and reliable identification method of herbal medicines than the traditional ones, which had a very broad application prospect.

## 4. Conclusion

A supervised pattern recognition method based on PLSDA algorithm by HPLC and NIR has been established to study and identify the information of herbal medicines. In addition, it was clarified from the results that, other than PCA and LDA that can merely have good learning performance and do well in the training sets, PLSDA model shows good performance in the area of identification of herbal medicines and can be perfectly employed in the analysis of the influence of different collection parts, collection time, different origins, and various species belonging to the same genera of herbal medicines at one time. The results show that this recognition method is a promising approach for the identification of complex information of herbal medicines.

## Supplementary Material

The supporting information for chromatographic condition of *Hibiscus mutabilis* L. or *Berberidis radix* and the pattern recognition results of Berberidis radix by PCA, LDA and PLSDA.

## Figures and Tables

**Table 1:** A detailed list of all samples information of *Hibiscus mutabilis* L. and different species of *Berberidis radix*.

Names	Sample group	Origins	Collection parts	Collecting time
*Hibiscus mutabilis* L.	1-1	Nanning, Guangxi	Leaves	2012.7
	1-2	Nanning, Guangxi	Stems	2012.7
	1-3	Nanning, Guangxi	Leaves and stems	2012.7
	1-4	Anhui	Leaves	2012.10
	1-5	South-Central University for Nationalities, Wuhan	Leaves	2011.11
	1-6	South-Central University for Nationalities, Wuhan	Leaves	2012.6
	1-7	South-Central University for Nationalities, Wuhan	Leaves	2012.8
	1-8	South-Central University for Nationalities, Wuhan	Fallen leaves	2012.12

*Berberidis radix *				
BSS	2-1	Jianshi, Hubei	Roots	2012.5
BHS	2-2	Jianshi, Hubei	Roots	2012.6
BGS	2-3	Jianshi, Hubei	Roots	2012.6
BTF	2-4	Jianshi, Hubei	Roots	2012.6

**Table 2:** A detailed list of *Hibiscus mutabilis* L. and *Berberidis radix* information.

Names	Sample group code	Group	Symbol	HPLC training samples	HPLC prediction samples	NIR training samples	NIR prediction samples
*Hibiscus mutabilis* L.	f1	1-1	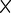	1st–3rd	1st–3rd	1st–50th	1st–30th
	f2	1-2	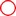	4th–6th	4th–6th	51st–95th	31st–65th
	f3	1-3	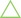	7th–10th	7th-8th	96th–147th	66th–93rd
	f4	1-4	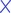	11th–13th	9th–11th	148th–201st	94th–119th
	f5	1-5	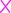	14th–18th	12th	202nd–255th	120th–145th
	f6	1-6	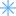	19th-20th	13th–16th	256th–312th	146th–168th
	f7	1-7	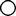	21st-22nd	17th–20th	313th–366th	169th–194th
	f8	1-8	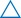	23rd–26th	21st-22nd	367th–421st	195th–219th

*Berberidis radix *	f1	2-1	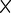	1st–3rd	1st–3rd	1st–50th	1st–30th
	f2	2-2	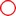	4th–6th	4th–6th	51st–95th	31st–65th
	f3	2-3	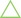	7th–10th	7th-8th	96th–147th	66th–93rd
	f4	2-4	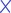	11th–13th	9th–11th	148th–201st	94th–119th
