# Supplementary material for: Rapid Discrimination for Traditional Complex Herbal Medicines from Different Parts, Collection Time, and Origins Using High-Performance Liquid Chromatography and Near-Infrared Spectral Fingerprints with Aid of Pattern Recognition Methods
Source: J Anal Methods Chem. 2015 Aug 9;2015:727589. doi: 10.1155/2015/727589 (PMC4546757; doi:10.1155/2015/727589)
Supplement: Supplementary file 1 — The supporting information for chromatographic condition of Hibiscus mutabilis L. or Berberidis radix and the pattern recognition results of Berberidis radix by PCA, LDA and PLSDA. [file 727589.f1.doc]

1.**Table S1**: Chromatographic condition for Hibiscus mutabilis L

| Time(min) | 0 | 10 | 35 | 55 |
| --- | --- | --- | --- | --- |
| Methano | 5 | 5 | 45 | 80 |
| 0.1% acetic acid | 95 | 95 | 55 | 20 |

2. **Table S2**: Chromatographic condition for Berberidis radix

| Time(min) | methanol | 0.25% phosphoric acid | water |
| --- | --- | --- | --- |
| 0 | 5 | 95 | 0 |
| 7 | 5 | 95 | 0 |
| 10 | 30 | 70 | 0 |
| 35 | 35 | 65 | 0 |
| 50 | 45 | 55 | 0 |
| 51 | 10 | 0 | 90 |
| 61 | 10 | 0 | 90 |
| 62 | 5 | 95 | 0 |
| 72 | 5 | 95 | 0 |

3. **Figure S1**: Results by PCA


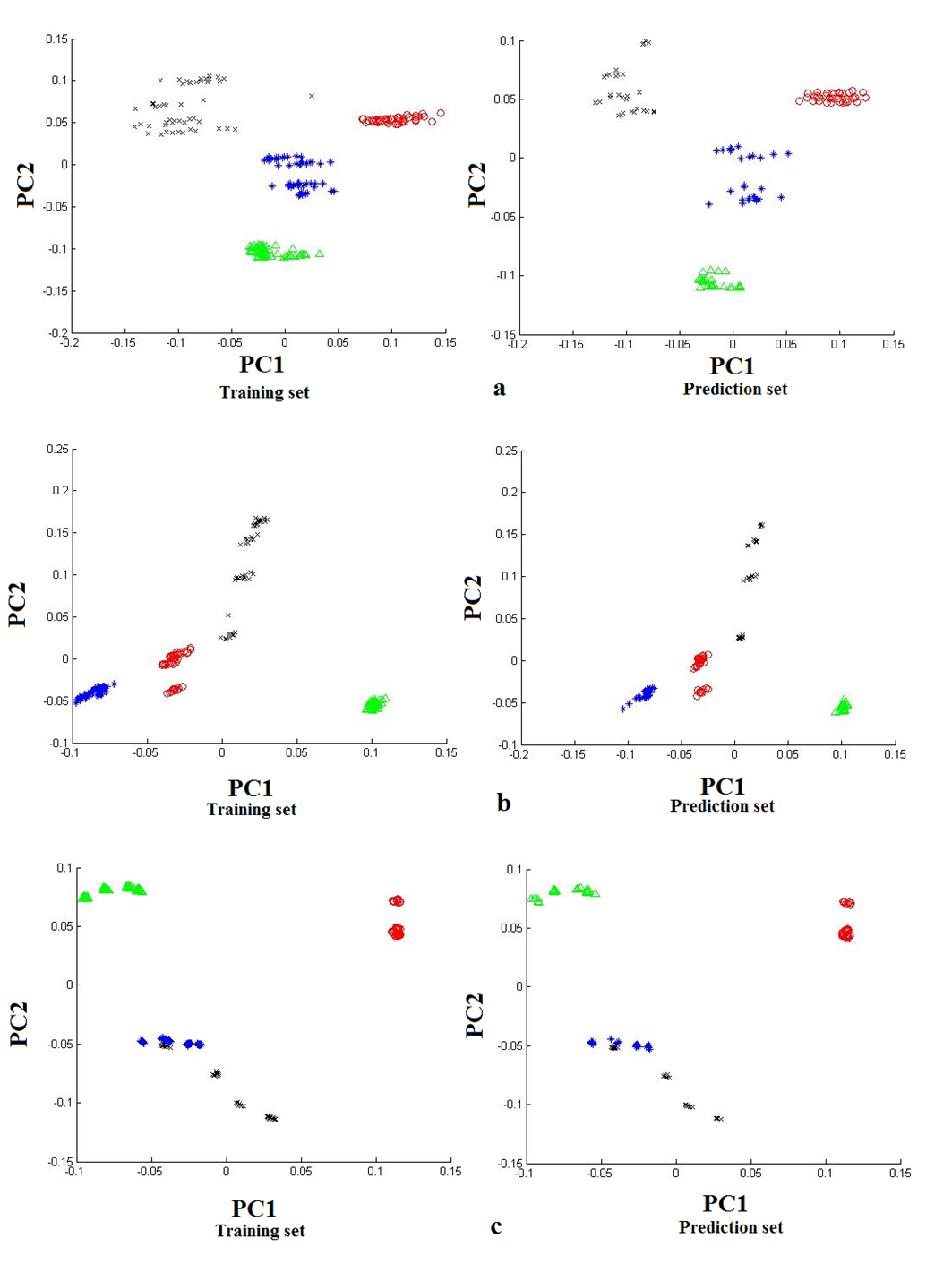


**Figure S1**: Score plots of the raw (a), MSC (b) and 2nd derivative (c) NIR spectra by PCA of 4 different kinds of Berberidis radix samples in training sets and in prediction sets.

4. **Figure S2:** Results by LDA


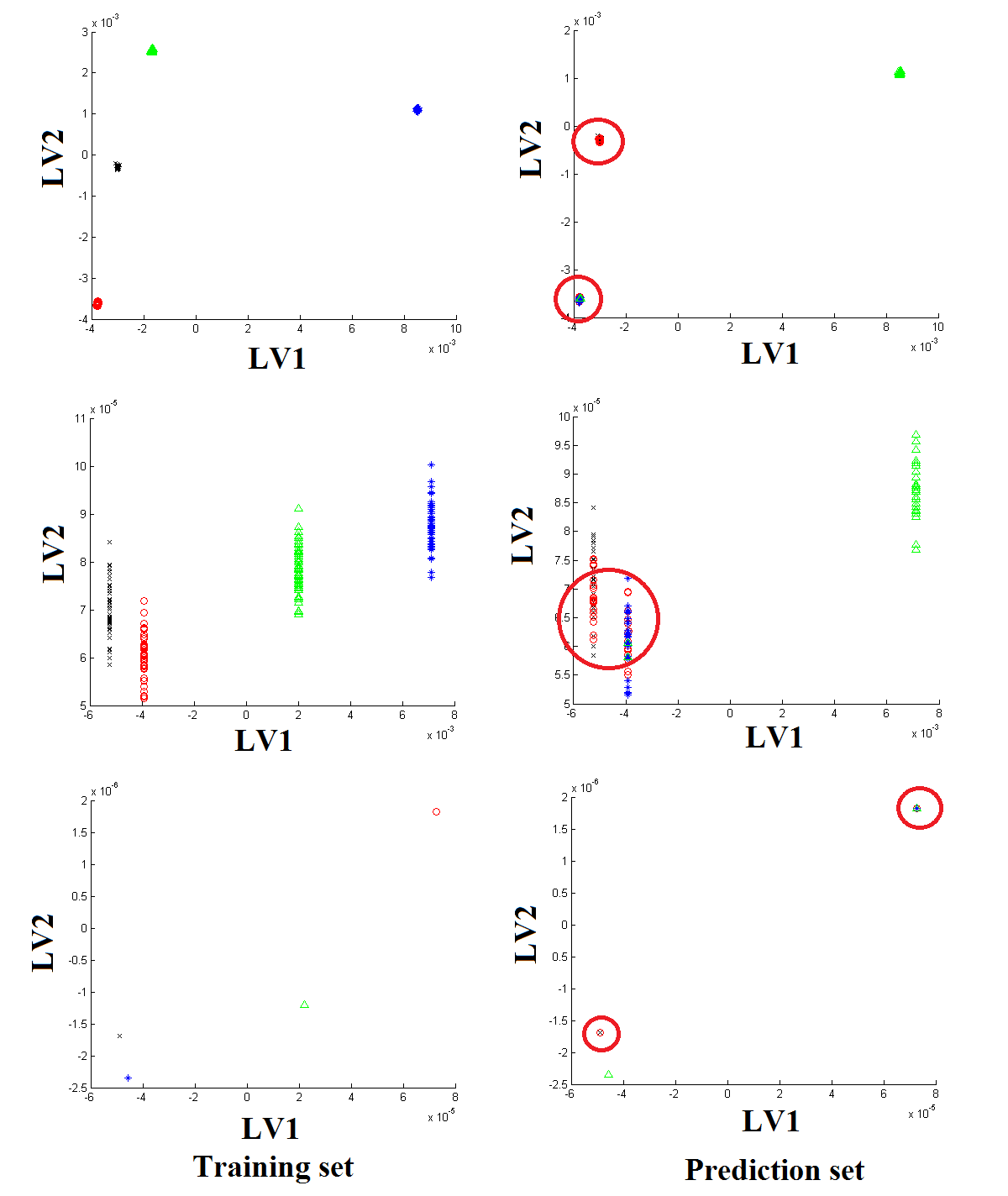


**Figure S2**: Score plots of the raw (a), MSC (b) and 2nd derivative (c) NIR spectra by LDA of 4 different kinds of Berberidis radix samples in training sets and in prediction sets.

5. **Figure S3:** Results by PLSDA


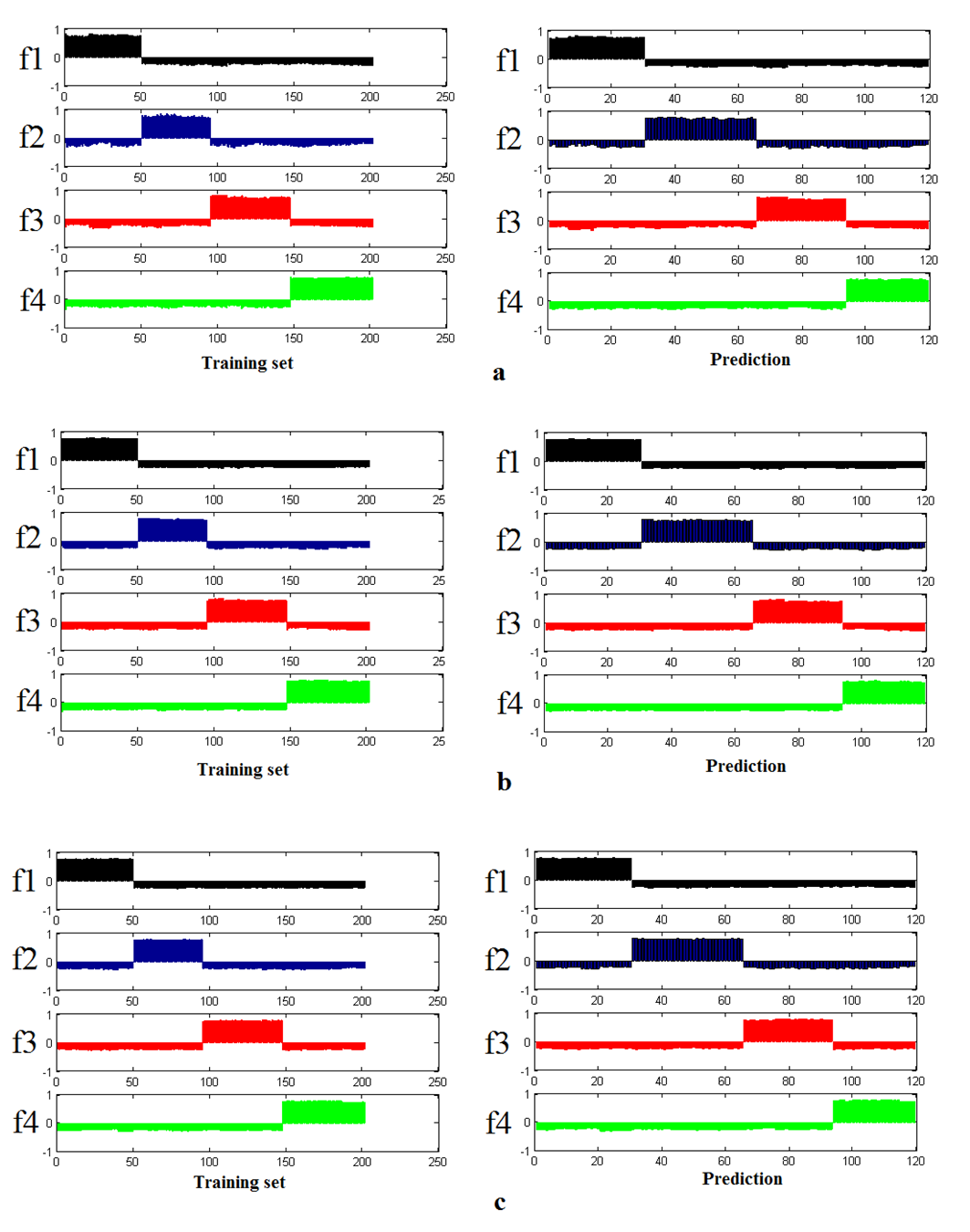


**Figure S3**: Assigned plots of dummy codes of the training set and prediction set for the raw (a), MSC (b) and 2nd derivative (c) NIR spectra in PLSDA model.
